# Editorial: Advancing understanding of developmental coordination disorder: diagnosis, mechanisms, and interventions

**DOI:** 10.3389/fnhum.2026.1954668

**Published:** 2026-09-15

**Authors:** Jorge Lopes Cavalcante Neto, Lemke Dorothee Jelsma, Tatiane Targino Gomes Draghi

**Affiliations:** 1 Department of Human Sciences, Bahia State University, Jacobina, Brazil; 2 Faculty of Behavioural and Social Sciences, Rijksuniversiteit Groningen, Groningen, Netherlands; 3 Faculty of Physical Therapy, Universidade Estadual do Norte do Parana, Jacarezinho, Brazil

**Keywords:** awareness, children, DCD, functioning, mechanisms

Developmental Coordination Disorder (DCD) is a complex and heterogeneous neurodevelopmental disorder affecting motor skills in approximately 6% of school-aged children; crucially, it is increasingly recognized not merely as an isolated motor deficit, but as a multifaceted condition with broad effects on daily functioning, cognition, and participation. It affects a substantial proportion of school-aged children, can persist into adulthood, and frequently co-occurs with other developmental conditions such as ADHD, autism spectrum disorder, learning disabilities, speech-language delays, and emotional or behavioral difficulties. This heterogeneity is now one of the defining features of DCD, helping explain why children can present with different motor, cognitive, and psychosocial profiles.

To help address these complexities, the Research Topic *Advancing understanding of developmental coordination disorder: diagnosis, mechanisms, and interventions* was launched in *Frontiers in Human Neuroscience* and edited by Dr. Jorge Lopes Cavalcante Neto and Dr. Dorothee Jelsma, with the collaboration of Dr. Tatiane Targino Gomes Draghi. Through the collaborative efforts of this editorial team and the contributing authors, eight articles were published as part of this Research Topic, bringing together studies that deepen the field's understanding of the etiology of DCD, how it is expressed in the brain, how functional reorganization and structural changes may facilitate behavioral improvement through optimized internal models and enhanced cognitive-motor coupling through effective interventions. At the same time, evidence from adulthood shows that altered motor-system physiology can remain present beyond childhood, supporting the view that DCD is a lifespan condition rather than a transient childhood difficulty (He et al., 2018).

Regarding diagnosis and underlying mechanisms, the studies by Song et al., Napoli et al., and He et al. (2018) identified both structural and functional brain abnormalities in children with DCD, providing further evidence for the neural basis of the motor impairments characteristic of the disorder. Together, these studies strengthen the evidence that children with DCD exhibit atypical patterns of brain activation and connectivity. Specifically, Napoli et al. demonstrated reduced activation across multiple brain regions, including Dorsolateral Prefrontal Cortex (DLPFC), Right Inferior Frontal Gyrus (rIFG), Posterior Cerebellum, and Supplementary Motor Area (SMA). Such neurofunctional alterations can have an impact on executive functions, attention, and cognitive control during motor tasks. These disruptions further compromise inhibitory control mechanisms and the regulation of motor responses, while also impairing the fine-tuning, adaptive motor control, and coordination of complex movements. Finally, they can make the planning, sequencing and execution of self-initiated voluntary movements difficult. Together, these multi-level deficits culminate in a complex functional phenotype that spans both motor performance and cognitive-attentional domains.

Song et al. assessed brain activity utilizing electroencephalography (EEG) in boys with DCD. At rest, functional connectivity was significantly lower across all analyzed frequency bands. Furthermore, during a visuomotor task, these children exhibited reduced spectral power in frontocentral and parietal regions, alongside an abnormal lateralization of beta-band connectivity around the motor cortex. This indicates a global deficit in spontaneous communication and synchronization across different neural networks. Functionally, this can impair the brain's forward planning, organize, and integrate sensorimotor information even before a movement is initiated, thereby disrupting the processing of motor preparation and initiation (feedforward) commands. Furthermore, it points to spatial processing difficulties and an inability to internally update body position in space during coordinated movement, ultimately resulting in a lack of mechanical precision and a significantly higher cognitive effort to execute motor tasks that should otherwise be automated. In young adults, He et al. (2018) demonstrated a reduction in interhemispheric cortical inhibition. This neurophysiological finding is highly consistent with the bimanual control difficulties, mirror movements, and motor modulation deficits that can persist well beyond childhood.

Despite these complex neural alterations, the findings also reinforce the encouraging message that children with DCD can improve substantially when support is structured, targeted, and sustained, as demonstrated in the intervention studies conducted by Pranjić et al., Ferreira et al., Ji et al., and Gao et al. Using an auditory rhythm-based intervention, Pranjić et al. found that children with DCD improved not only their motor performance but also their rhythmic abilities. Together with EEG-based investigations of brain function in DCD, this auditory rhythm-based intervention points to the possibility that timing, sensory integration, and motor coordination are deeply interconnected, opening the door to interventions that use rhythm, sound, and movement as complementary therapeutic tools (Song et al.). Given the sensory processing difficulties commonly observed in DCD, interventions incorporating music education and auditory–motor training may represent promising therapeutic approaches.

Similarly, Ferreira et al. compared aquatic- and land-based intervention protocols and reported significant improvements in motor performance among children with DCD. Notably, the intervention effects were comparable in magnitude to those observed in typically developing peers and were maintained over time. Complementing these findings, Ji et al. highlighted the importance of structured motor interventions for improving motor coordination in children with DCD. Their findings suggest that such interventions may improve not only core motor skills, but also executive function, participation, engagement, and quality of life. Furthermore, the neuroimaging results suggest that these gains can be accompanied by neuroplastic changes in connectivity and white-matter organization. Gao et al. expands on this reasoning by presenting DCD as a motor-cognitive disorder in which deficits in movement anticipation, motor learning, and cognitive control contribute to daily life difficulties. In this context, strategies such as cognitive orientation to daily occupational performance (CO-OP), motor imagery and action observation emerge as relevant avenues for improving memory, attention, mental planning, and occupational performance.

Finally, the importance of increasing awareness of DCD was highlighted by van Abswoude et al., who used a qualitative approach to explore the perspectives of stakeholders within the Dutch school system. Participants reached a clear consensus regarding the need to improve awareness of DCD and recognized their own responsibility in promoting knowledge dissemination and supporting children with the disorder.

Collectively, the studies included in this Research Topic advance our understanding of the neural mechanisms underlying DCD, provide evidence supporting innovative intervention approaches, and emphasize the critical importance of raising awareness across educational and healthcare settings. Together, these contributions represent an important step toward improving the identification, management, and long-term outcomes of individuals with DCD.
